# Interdisciplinary Management of Open Interproximal Embrasures

**DOI:** 10.1111/jerd.70015

**Published:** 2025-08-03

**Authors:** Yudai Ogawa, Otto Zuhr, Gustavo Avila‐Ortiz, Yukuhiro Kosaka, Tomohiro Ishikawa, Yi‐Wen Cathy Tsai, Dean Morton, Toshiki Nagai

**Affiliations:** ^1^ Dentist, Private Practice Shizuoka/Tokyo Japan; ^2^ Department of Dentistry and Oral Surgery Keio University School of Medicine Tokyo Japan; ^3^ Dentist, Private Practice Munich Germany; ^4^ Department of Periodontology, Center of Dentistry and Oral Medicine (Carolium) Johann Wolfgang Goethe‐University Frankfurt/Main Germany; ^5^ Department of Oral Medicine, Infection, and Immunity, Harvard School of Dental Medicine Harvard University Boston Massachusetts USA; ^6^ Department of Molecular and Regenerative Prosthodontics Tohoku University Graduate School of Dentistry Sendai Miyagi Japan; ^7^ Dentist, Private Practice Shizuoka Japan; ^8^ Department of Periodontology, School of Dentistry Tri‐Service General Hospital and National Defense Medical Center Taipei Taiwan; ^9^ Center for Implant, Esthetic and Innovative Dentistry Indiana University School of Dentistry Indianapolis Indiana USA; ^10^ Department of Prosthodontics Indiana University School of Dentistry Indianapolis Indiana USA

**Keywords:** esthetic dentistry, interdental papilla, open interproximal embrasures, periodontal regeneration

## Abstract

**Objectives:**

To describe key etiological and risk factors associated with open interproximal embrasures (OIE) and propose a systematic interdisciplinary approach for their management, emphasizing the integration of periodontics, orthodontics, and prosthodontics.

**Overview:**

Optimal esthetics and function are priority outcomes in contemporary dentistry, often necessitating an interdisciplinary approach to achieve satisfactory results. OIE, commonly referred to as “black triangles,” result from the loss of interdental papillary height due to a variety of factors such as unfavorable bone crest‐to‐contact point distance, interproximal width, gingival thickness, tooth form, and axial root inclination. Their prevalence and clinical presentation may be influenced by age, history of orthodontic or periodontal treatment, and tooth morphology. Comprehensive diagnostic protocols and treatment strategies for the management of OIE may involve periodontal regeneration, soft tissue augmentation, orthodontic alignment, and restorative procedures, as demonstrated in this article.

**Conclusion:**

A structured, patient‐specific interdisciplinary approach emphasizing both “pink” (soft tissue) and “white” (tooth structure) esthetics is essential for effectively treating OIE, ultimately improving patient satisfaction and quality of life. Future clinical research should focus on evaluating the efficacy of various interdisciplinary treatment strategies.

**Clinical Significance:**

This article underscores the importance of interdisciplinary collaboration in managing OIE to optimize esthetics and function, ultimately enhancing patient satisfaction and quality of life.

## 1 Introduction

In the ever‐evolving landscape of modern dentistry, the perception and relevance of smile esthetics have undergone a profound transformation over the past decade. Nowadays, patients prioritize the visual appeal of their smiles more than ever. Nonetheless, this shift towards esthetics does not diminish the essential role of function. Achieving both optimal function and esthetics often requires the integrated expertise of multiple dental specialties, including periodontics, orthodontics, and prosthodontics [1]. Interdisciplinary dentistry is no longer just a fancy trend but a strategic imperative for optimizing the outcomes of therapy in a predictable manner.

A meticulous assessment and coordinated consideration of the “white” and “pink” components are essential to ensuring optimal esthetic outcomes. Specific criteria for the management of cases involving smile esthetics have been introduced in the literature, encompassing elements such as tooth form, gingival/mucosal architecture, and interdental papilla features [2]. The morphology of the interdental papilla holds significant importance for periodontists, orthodontist, prosthodontists, restorative dentists, and their patients [3]. Its absence can result in cosmetic problems, commonly referred to as “black triangle disease,” along with phonetic issues related to the passage of air or saliva, and an increased susceptibility to food impaction [4, 5]. It has been reported that 100% of females and 95% of males display a variable amount of papillary tissue when smiling [6]. Objective assessments have also revealed that orthodontists recognize a papillary loss of 2 mm as an esthetic deficiency, while the general population perceives it when the loss is at least 3 mm [7].

Open interproximal embrasures (OIE) are clinical entities that result from the loss of interdental papillary height due to a variety of factors. Burke and collaborators reported a prevalence of OIE in 15% of a general adolescent population, 41.9% among adolescents after orthodontic therapy for maxillary incisor crowding, and approximately 38% in adults after orthodontic therapy [5, 8]. These findings suggest that orthodontic treatment may be one of the risk factors for the development of OIE. Age has also been associated with wider and taller embrasure spaces in adults [9]. OIE are found in 67% of individuals aged 20 and above, in contrast to just 18% in those under the age of 20 [10]. This phenomenon may be explained by the thinning of the oral epithelium, decreased keratinization, and reduced papilla height associated with aging [11]. Additionally, a common sequela of periodontitis is loss of interdental papillary height due to underlying alveolar bone loss [9]. As described in previous studies, a history of periodontal surgery involving elevation of the interdental papilla can lead to significant loss of height [12, 13, 14].

This article aims to discuss key etiological and risk factors associated with the onset and progression of OIE, and propose a systematic interdisciplinary approach for their management.

## 2 Risk and Etiologic Factors

**FIGURE 1:**
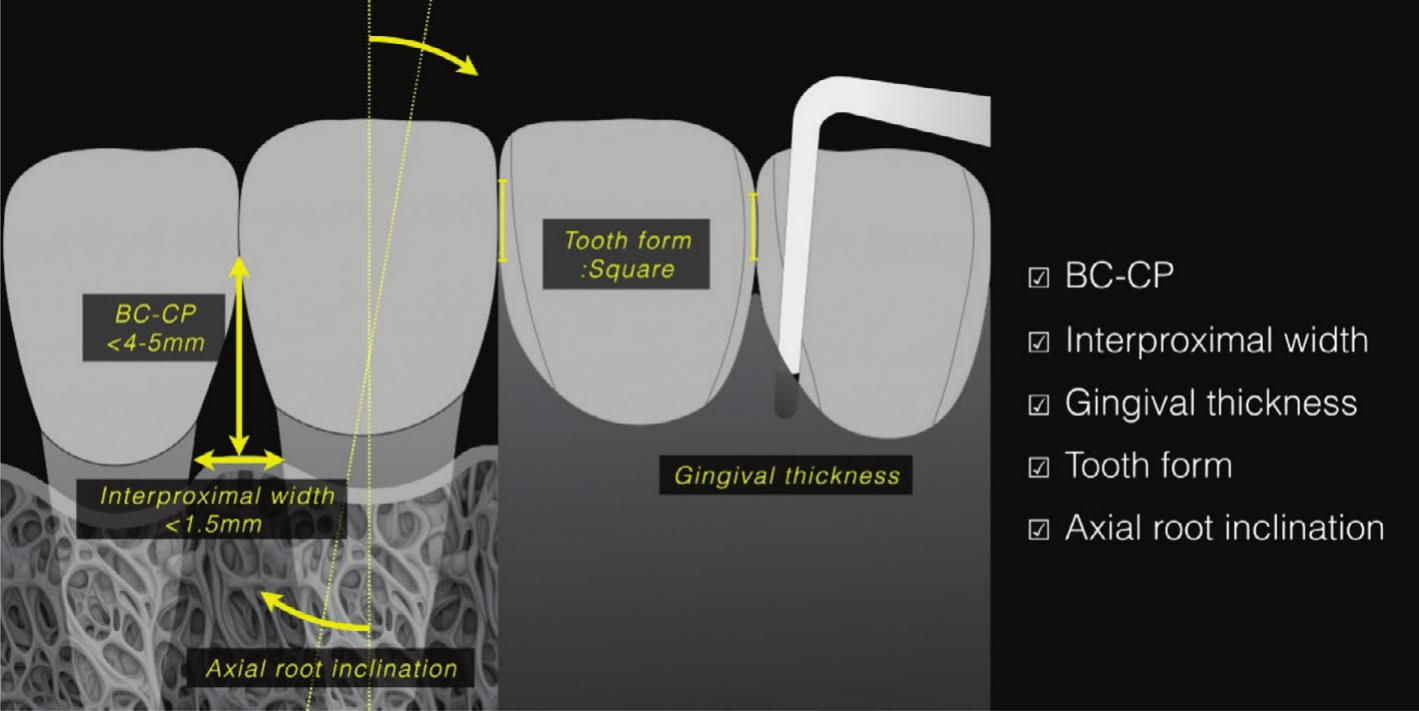
Five key factors related to the interdental papilla architecture.

Before initiating treatment, it is essential to analyze key factors that may influence the onset and progression of OIE (Figure 1).

### 2.1 Bone Crest‐to‐Contact Area Distance

It is well established that as the distance between the bone crest and the most apical point of the contact area (BC‐CP) increases, the likelihood of having optimal interproximal papillary fill decreases. In a landmark study, Tarnow and coworkers reported that when BC‐CP height is ≤ 5 mm, complete papillary fill occurs in nearly 100% of sites; however, as this distance increases, the likelihood of incomplete fill rises accordingly [15]. These findings align with those from other similar investigations [16, 17, 18]. In contrast, Kolte et al. observed that a height of ≤ 4 mm is required to have a complete papillary fill [19], which is a more stringent threshold compared to other studies [15, 16, 17, 18]. This numerical discrepancy may be explained by methodological differences, as Kolte et al. made direct measurements after elevating a flap [19]. Nevertheless, the available evidence consistently indicates that the greater the BC‐CP, the lower the chance of having complete interdental papillary fill. Variables associated with greater BC‐CP include: (1) interdental contact areas shifted coronally due to poor dental restorations or dental misalignment, and (2) interdental bone crests shifted apically as a result of interproximal bone loss.

### 2.2 Interproximal Width

Interproximal width is the distance between tooth roots at the level of the interdental bone crest. A discernible negative correlation appears to exist between BC‐CP height and interproximal width. In a study conducted by Cho et al., it was found that in order to ensure adequate papillary height, an interproximal width of ≤ 1.5 mm is preferable when BC‐CP is ≤ 5 mm, while a range of 2.0–2.5 mm is advisable when BC‐CP is ≤ 4 mm [20]. Similar observations have been reported by other investigators [19, 21]. This relationship can be explained by the findings of Kurth and Kokich, which suggest that an interproximal embrasure area of > 5.09 mm^2^ is correlated with the presence of OIE [5]. It is also important to emphasize that interproximal width is also intricately linked to tooth form and axial root inclination.

### 2.3 Gingival Thickness

Although it has been reported that the faciolingual thickness of the interproximal papilla does not seem to be a critical factor influencing the presence of complete papillary fill, there is convincing evidence indicating that thicker interproximal gingival tissue increases the likelihood of achieving optimal papillary fill [22]. Chow et al. found that the mean interproximal gingival thickness was greater when the papilla was complete compared to when it was deficient, measuring 1.5 versus 0.87 mm, respectively (*p* < 0.01) [17]. Furthermore, Joshi et al. reported that when interproximal gingival thickness was > 1 mm, 94.5% of sites exhibited complete papillary fill, whereas only 32.5% of cases demonstrated complete papillary fill when gingival thickness was ≤ 1 mm [18]. Among different options to surgically enhance gingival thickness, those involving the use of autogenous connective tissue grafts (CTG) remain the gold standard. CTG not only increases thickness, but also offers the potential for creeping attachment [23].

### 2.4 Tooth Form

In general, square‐shaped teeth and long contact areas are favorable conditions for achieving complete papillary fill. These features correlate with BC‐CP and interproximal width. Square‐shaped teeth often present shorter interproximal widths and longer contact areas, which contribute to a reduced BC‐CP [24]. Conversely, triangular or irregular tooth shapes and crowding are generally unfavorable [25]. Nevertheless, clinicians must carefully consider the delicate balance among all relevant factors to avoid compromising the overall natural appearance of teeth. Kokich et al. found that an interproximal contact area length exceeding 1.5 mm beyond the standard could be perceived as unnatural by laypersons [7]. Therefore, when planning a modification of tooth form to enhance papillary fill, it is recommended to position the most apical point of the contact area at 4–5 mm from the alveolar crest, with a crown width (CW) to crown length (CL) ratio of less than 0.87 [17]. This approach contributes to creating a natural tooth form in prosthodontic or restorative procedures. In orthodontics, interproximal reduction (IPR) is a method used to enhance tooth shape and create space to facilitate tooth movement. Typically, IPR involves the removal of approximately 0.25–0.75 mm of enamel. This process, coupled with space closure, typically shifts the most apical point of the contact area towards the gingival region, which decreases BC‐CP [26].

### 2.5 Axial Root Inclination

It has been reported that orthodontic treatment to straighten roots with an unfavorable axial inclination may contribute to eliminating or reducing the dimensions of OIE [9]. For a successful outcome, a careful assessment of the relationship between the contact areas of adjacent teeth and their axial root inclination is fundamental. For example, when there is axial root inclination towards the interdental space, not only may the interproximal width be reduced but also the interdental gingiva is compressed, leading to potentially positive effects [16]. Mean axial root inclination in normal/closed interproximal embrasures has been reported to converge at 3.65° [5]. When the interproximal crown shape, BC‐CP, and contact area to incisal edge remain constant, a rise of 1° in root divergence elevates the likelihood of OIE occurrence by 14%–21% [5]. The orientation of tooth movement, along with the labiolingual thickness of the supporting bone and soft tissue, also influences the morphology of interproximal gingival tissue following orthodontic treatment. Lingual tooth movement typically results in thickening of the facial gingival tissue and an increase in papilla height [27]. Conversely, labial tooth movement causes the facial soft tissue to thin and move apically [27]. Additionally, anomalies in the vertical dimension may be relevant depending on the periodontal conditions (e.g., absence of gingival attachment). Teeth that are excessively proclined out of the dental arch are sometimes indicative of pathological tooth migration due to attachment loss; their restoration can be challenging or contraindicated. Even in the presence of healthy periodontal tissues, severely proclined teeth may induce the formation of contact areas with unfavorable configurations and locations (i.e., coronally displaced, which increases the BC‐CP).

## 3 Therapeutic Protocols and Recommendations

**FIGURE 2:**
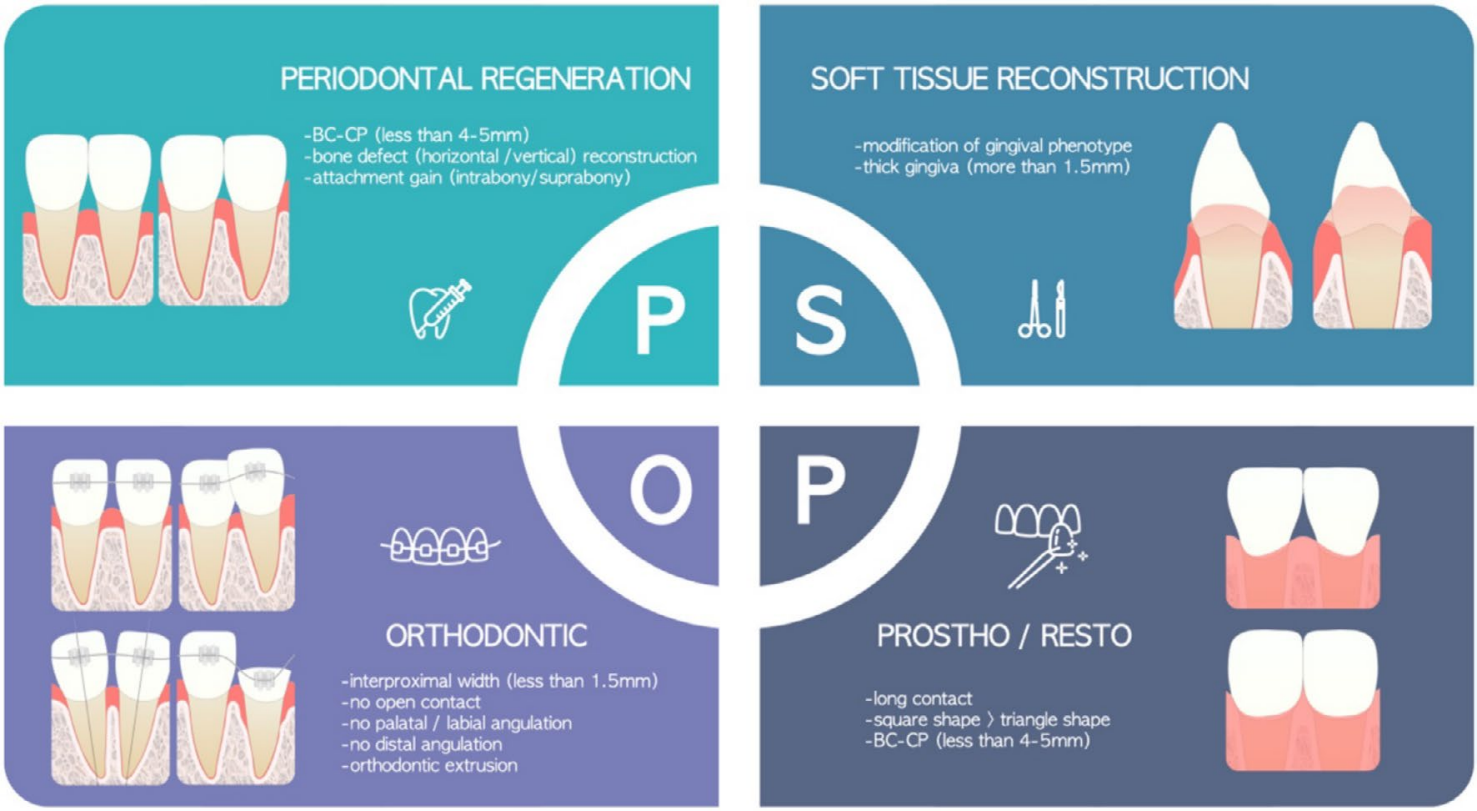
Four therapeutic modalities to manage OIE.

When attempting to manage OIE by restoring the morphology of deficient papillae or by creating the illusion of complete papillary fill, it is essential to meticulously assess the five key factors addressed in the previous section. Following this assessment, the desired outcomes should be clearly identified, and a precise treatment plan should be developed. Depending on the etiology, there are essentially four treatment approaches available to manage OIE: periodontal regeneration, interproximal soft tissue augmentation, orthodontic treatment, and prosthetic/restorative therapy (Figure 2).

### 3.1 BC‐CP

Unfavorable BC‐CP is frequently observed when (a) the contact area is coronally shifted due to ill‐shaped dental restorations or tooth misalignment, and/or (b) the interdental bone crest is apically shifted (e.g., due to periodontitis). In the first situation, restorative and/or orthodontic therapy should be considered. As a general guideline, it is recommended to establish the features of the ideal contact area, whose most apical end should be located at approximately 4–5 mm from the alveolar crest, with a CW to CL ratio of less than 0.87, as this contributes to recreating a natural tooth form [17]. Additionally, it is important to recognize that the contact length‐to‐tooth height ratio varies among the maxillary anterior teeth: 50% for the central incisor, 40% for the lateral incisor, and 30% for the canine [28]. Contact length refers to the vertical dimension of the restorative material that connects adjacent units in a dental prosthesis, contributing not only to structural integrity but also to the overall esthetic outcome. The decision to use veneers, crowns, or direct restorations depends on factors such as the occlusal relationship and the extent of the remaining tooth structure. As such, a collaborative discussion between the clinician and the patient is essential to determine the most appropriate treatment approach.

In the latter situation, a surgical approach to gain soft tissue may be considered. However, there are currently no predictable surgical techniques to augment the papilla mainly due to the fragility and low blood supply that typically characterizes this structure [16]. However, a few reports have discussed supracrestal soft tissue attachment gain and reconstruction, suggesting that these approaches may be worth considering. Cases involving supragingival attachment regeneration (SAPRE) [29], the apically incised coronally advanced surgical technique (AICAST) [30], and the tunnel wall approach [31] have been reported. Performing vertical tissue augmentation in narrow areas like interdental papillae remain challenging; thus, methods that avoid papillary incisions are generally more reliable and eliminate the risk of tissue dehiscence while allowing for tissue reconstruction of the alveolar crest. Depending on the type of bone loss present, different approaches may be considered for treatment. In general, bone loss exhibits two main patterns. First, if there is a vertical defect with a predominant infrabony component, periodontal regeneration should be considered. However, as noted by Tal, et al., periodontal surgery in the interdental space may lead to scar formation in the soft tissue, the disappearance of the col. beneath the interdental contact area, and the reorganization of collagen fiber groups [13]. Thus, techniques that avoid incisions in the interdental papilla, such as the entire papilla preservation (EPP) [32], double‐sided EPP (DEPP) [33], and non‐incisional papilla‐sparing approach (NIPSA) [34, 35] may be effective. Conversely, there is currently no surgical approach that can predictably increase the bone crest height, if horizontal crestal bone resorption without or with a minimal infrabony component is present.

In addition to these surgical and restorative strategies, orthodontic extrusion (OE) may serve as a valuable adjunctive or alternative treatment modality in select cases. When applied appropriately, OE has the potential to correct unfavorable bone crest‐to‐contact point relationships, as well as regenerate or improve periodontal bony and papillary defects by enhancing the vertical dimension of the supporting structures [36]. This biologically driven approach can help establish a more favorable esthetic and functional outcome in challenging cases.

### 3.2 Interproximal Width

To achieve a favorable interproximal width compatible with complete papillary fill, orthodontic treatment may be considered. While it is rare to perform orthodontic treatment solely for the enhancement of the papilla, such intervention may be pursued in cases where malocclusion adversely affects the papillary morphology or when there is excessive interdental distance, causing a patent diastema. Orthodontic treatment can also be used to improve axial root inclination, and interproximal stripping can be used to reposition the most apical point of the interproximal contact area (CP), creating a longer contact area, thereby improving interproximal width.

### 3.3 Gingival Thickness

Soft tissue augmentation using CTG is an effective method for improving gingival thickness [23]. Autogenous soft tissue grafts can be harvested from various sites, such as the palatal mucosa, tuberosity, or edentulous regions, each offering distinct advantages and limitations depending on the clinical scenario [37]. While autogenous grafts are considered the gold standard due to their biocompatibility and predictable outcomes, recent advancements have highlighted the effectiveness of substitutes, such as allografts or xenografts. These alternatives, particularly when combined with biologics (e.g., recombinant human growth factors), have shown potential in promoting gingival thickening [38]. For example, regenerative therapies incorporating fibroblast growth factor‐2 (FGF‐2) hold promise for enhancing oral soft tissue regeneration [39]. The choice of technique depends on the specific clinical situation and requires careful consideration. For augmentation techniques in papilla reconstruction, one widely used method is the tunnel technique, which is noteworthy for its minimally invasive nature, as it accesses the buccal or lingual aspects without incising the marginal aspect of the papilla. Not only does this preserve papillary integrity, but it also facilitates the creation of a submucosal space for the application of tissue grafts or other materials with the purpose of coronally elevating the papilla [32, 33]. Another effective technique that does not involve incising the papilla is the palatal island flap [40].

### 3.4 Tooth Form

To achieve an ideal tooth form, prosthetic or restorative approaches may be indicated; however, these options should be considered after addressing other factors to attain an ideal natural appearance, as previously discussed.

### 3.5 Axial Root Inclination

Optimizing axial root inclination is best accomplished through orthodontic treatment guided by a comprehensive full mouth assessment and the patient's preferences. The primary goal when relying on orthodontic therapy should be to establish an ideal root axis alignment, although in some sites it may be beneficial for the papillary structure to have a slight root inclination towards the interdental space. While minor adjustments may be required in certain cases, excessive root inclination towards the interdental space should be avoided.

## 4 Clinical Scenarios

Before initiating treatment, it is essential to identify and control deleterious habits that may affect the course of therapy. Traumatic oral hygiene, especially the improper use of interdental brushes or floss, can contribute to the development of black triangles [41, 42]. To avoid this, it is necessary to provide guidance on appropriate oral hygiene practices [43].

In summary, evaluating the five factors and integrating the four different therapeutic modalities aforementioned are essential for formulating an effective treatment plan (Figures 1 and 2).

### 4.1 Scenario 1. Healthy Periodontium—Restorative Dentistry

**FIGURE 3:**
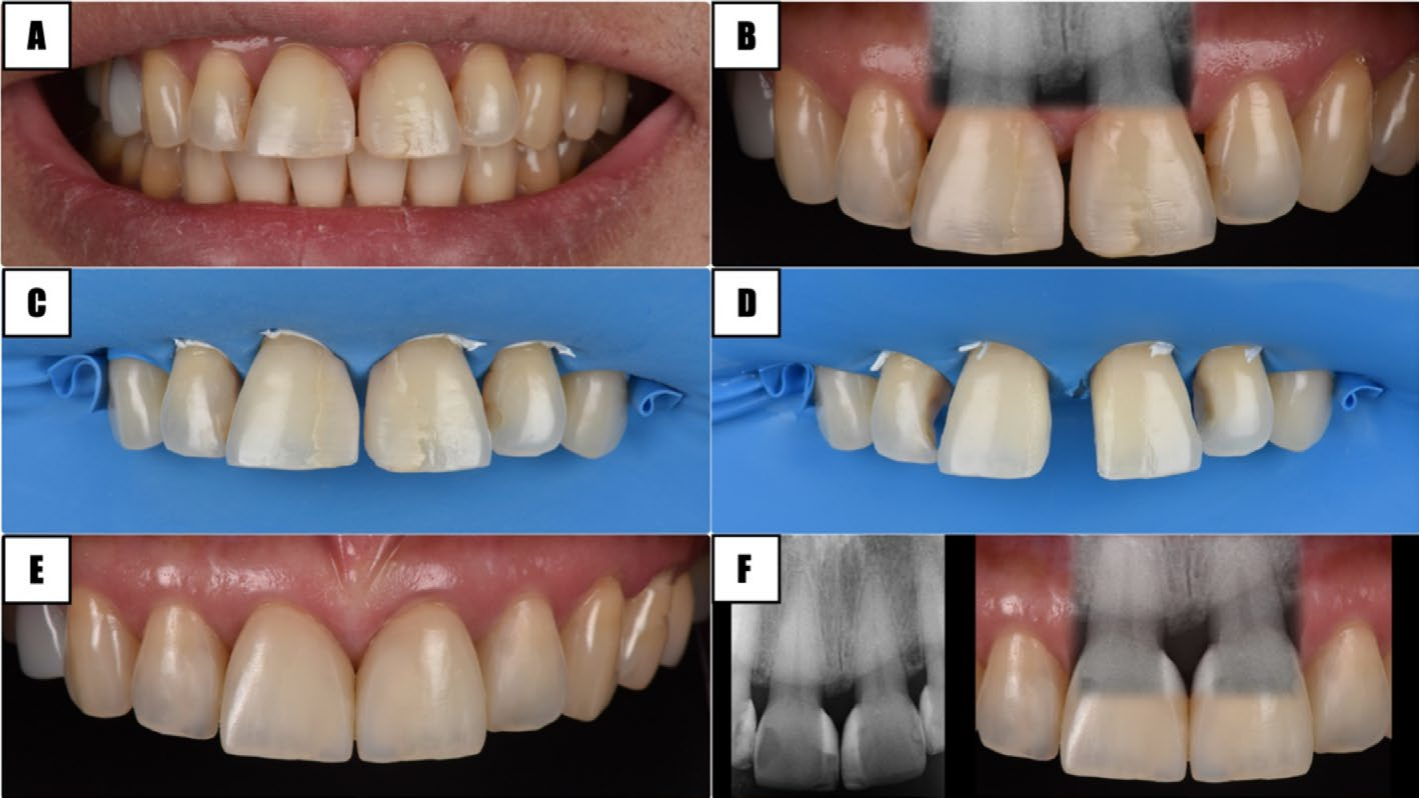
Scenario 1: healthy periodontal tissues—direct bonding. (A and B) Initial condition, (C and D) direct bonding procedure, (E and F) postoperative condition with periapical (PA) radiographs interposed.

A periodontally healthy patient exhibited an open embrasure upon smiling and expressed a desire for an esthetic enhancement (Figure 3A,B). The interdental papilla tip was slightly coronal to the level of the cementoenamel junction (CEJ). According to Cardaropoli et al.'s Papilla Presence Index (PPI), this case, where the papilla is not entirely present and lies apical to the contact area yet does not reveal the interproximal CEJ, would be classified as PPI 2 [44]. The following are specific diagnostic observations:

1. BC‐CP: greater than ideal.
2. Interproximal width: excessive, diastema present.
3. Gingival thickness: adequate (≥ 1 mm).
4. Tooth form: not ideal and lack of contact area.
5. Axial root inclination: normal.

According to the assessment of the five key factors, BC‐CP, interproximal width, and tooth shape are subjects of improvement. However, it was conveyed to the patient that the case could be treated without addressing the interproximal width. In further detail, full arch orthodontic treatment was not recommended due to the possibility of creating diastemas in other areas, even if the midline was closed with minor tooth movement. Treatment duration and cost were also factored in. To address BC‐CP and tooth shape, a direct restoration with bonded composite resin was the treatment of choice. First, the existing defective restorations were removed. Subsequently, under rubber dam isolation, the BC‐CP height was adjusted to approximately 4 mm, followed by direct bonding to achieve an esthetic restoration (Figure 3C,D). In this case, due to the large interproximal width, the BC‐CP distance was intentionally set shorter, resulting in an elongated contact area. This allowed for a natural finish by reshaping the teeth into a square form. The same protocol was followed between the central and lateral incisors (Figure 3E,F).

### 4.2 Scenario 2. Healthy Periodontium—Orthodontics

**FIGURE 4:**
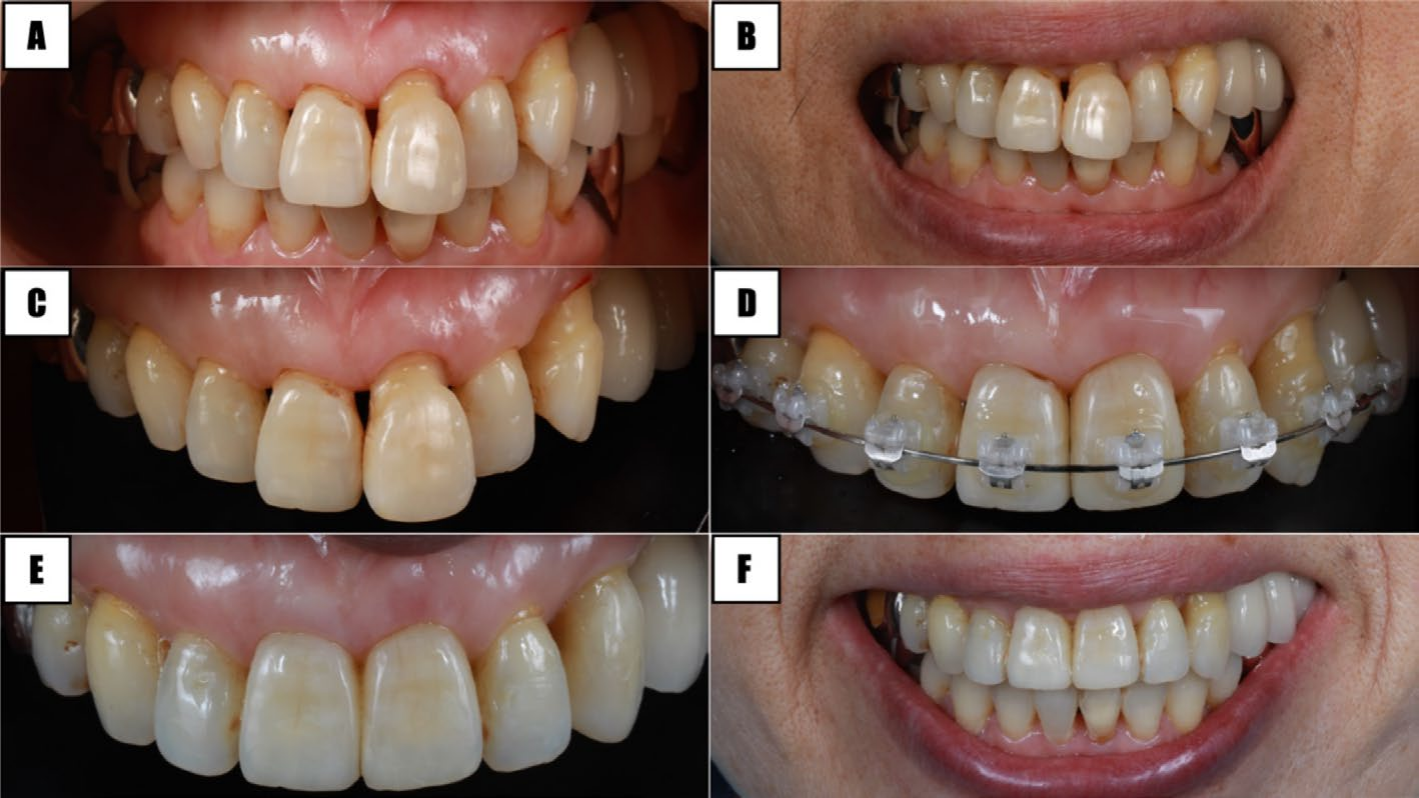
Scenario 2: healthy periodontal tissues—orthodontics. (A–C) Initial condition, (D) full arch orthodontic treatment, (E and F) postoperative condition.

Similar to the previous scenario, this patient presented an OIE in the midline, between the two central incisors and wanted to improve her esthetics (Figure 4A–C). Periodontal health was present, but the position of the interdental papilla was located apical to the CEJ (PPI 4) [44]. The following are specific diagnostic observations:

1. BC‐CP: Greater than ideal mainly due to tooth extrusion.
2. Interproximal width: excessive because of an abnormal tooth position.
3. Gingival thickness: adequate (≥ 1 mm).
4. Tooth form: not ideal.
5. Axial root inclination: abnormal.

Other than gingival thickness, all other diagnostic items were subjects of improvement. Orthodontic treatment was planned to correct the tooth extrusion and root inclination (Figure 4D). Then, tooth shape and interproximal distance were refined via enamel recontouring with polishing discs (Figure 4E,F).

### 4.3 Scenario 3. Healthy Periodontium—Reconstructive Periodontal Surgery and Restorative Dentistry

**FIGURE 5:**
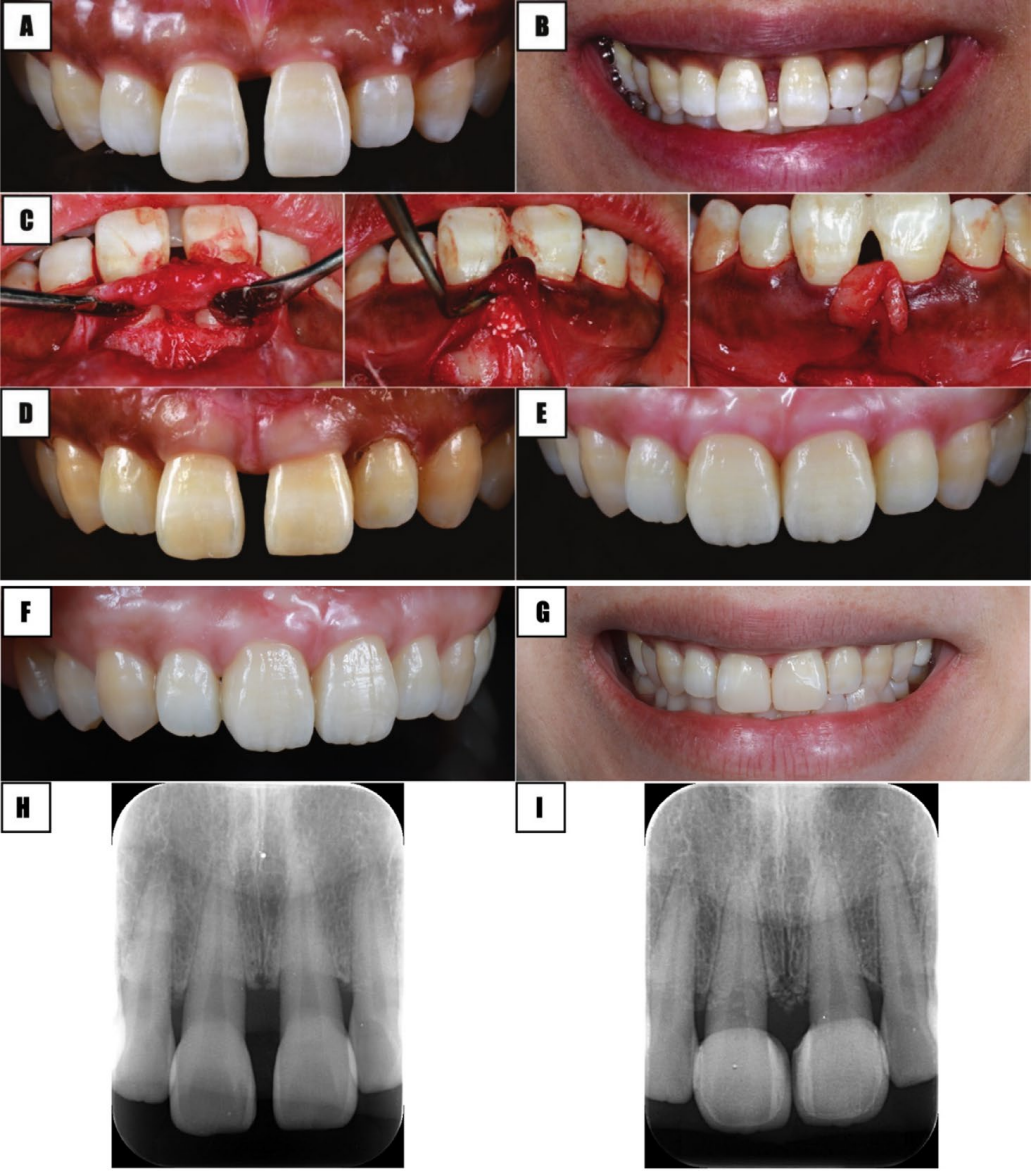
Scenario 3: healthy periodontal tissues—restoration and plastic surgery. (A and B) Initial intraoral condition, (C) periodontal surgery, (D) aspect after periodontal surgery, (E) frontal view after restorative treatment. (F) Veneer treatment and postoperative condition. (G) At 3 years post‐operation. (H) Initial periapical radiograph. (I) At 3 years follow up periapical radiograph.

This periodontally healthy patient presented a large diastema in the midline and desired to enhance her smile esthetics (Figure 5A,B). The interdental papilla tip was positioned apical to the CEJ, exhibiting a flat shape (PPI 3) [44]. The following are specific diagnostic observations:

1. BC‐CP: greater than ideal due to the apical position of the alveolar crest.
2. Interproximal width: excessive, presence of a diastema.
3. Gingival thickness: thin (< 1 mm).
4. Tooth form: not ideal and lack of contact area.
5. Axial root inclination: normal.

All items above are subject to improvement except axial root inclination. However, after careful analysis and discussion, Point 2 was not addressed for the same reasons as in Scenario 1. To improve points BC‐CP and gingival thickness, periodontal surgery was performed using the SAPRE technique, as described by Ogawa and collaborators in 2023 (Figure 5C) [29]. After 1 year, once the periodontal tissues were fully mature and stable (Figure 5D), the contact area and the tooth shape were modified with ceramic veneers to improve BC‐CP and the tooth form (Figure 5E). Radiographic results demonstrated an increase in opacity with bone substitute material (Figure 5E,F–I).

### 4.4 Scenario 4. Periodontitis Patient—Reconstructive Periodontal Surgery

Disclosure: the 2‐year outcomes of this case were published in a previous issue of this journal [29].

**FIGURE 6:**
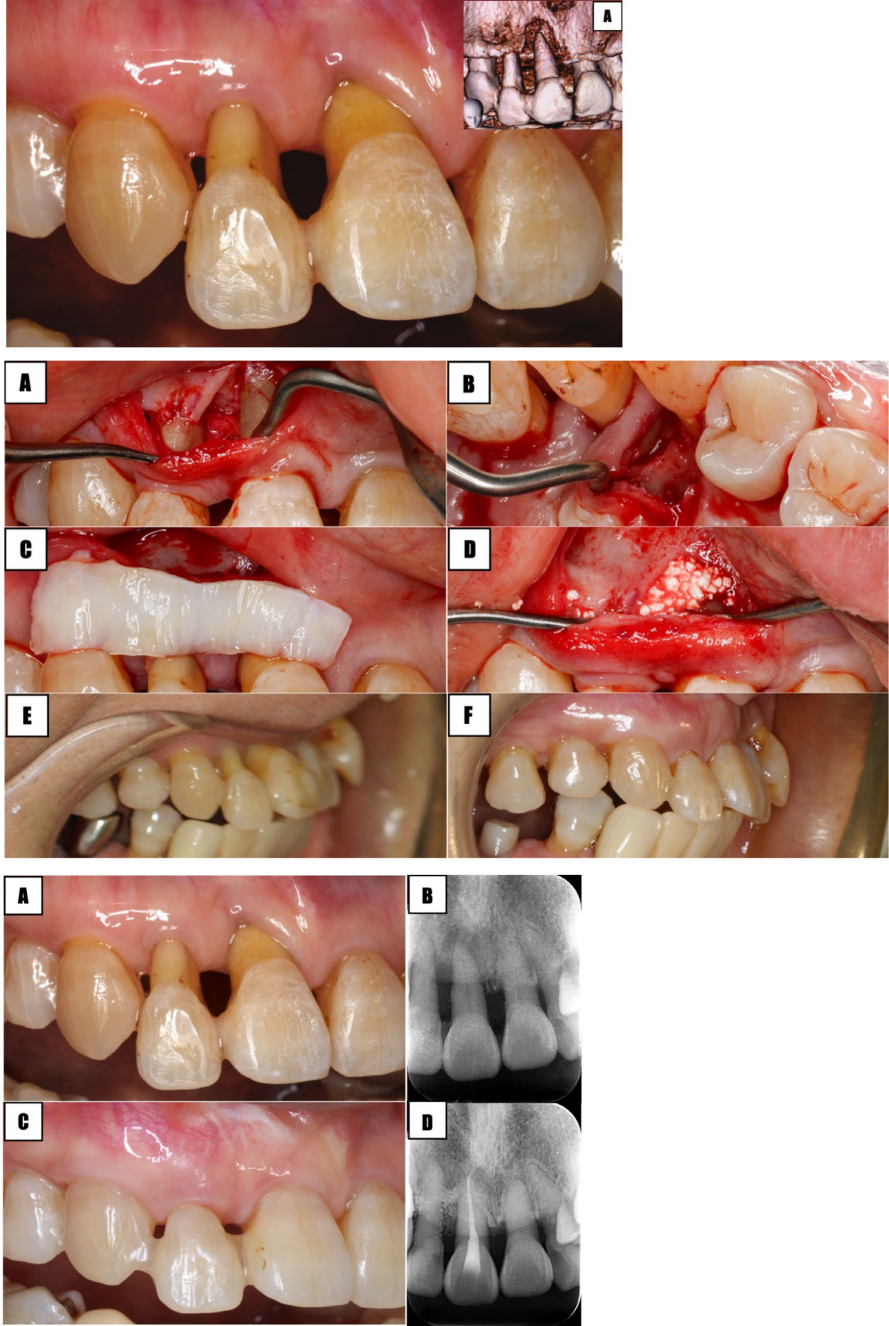
Scenario 4: unhealthy periodontal tissues. (A–D) SAPRE procedure, (E and F) pre‐postoperative condition from side view. (A and B) Initial intraoral condition and PA. (C and D) Clinical and radiographic status at 4 years post‐op after SAPRE.

This periodontitis patient exhibited several OIEs accompanied by gingival recession in the anterior maxilla (Figure 6). Because the papilla tip was apical to both the interproximal and buccal CEJ, the PPI was classified as 4 [44]. The patient was concerned about her periodontal health and esthetic appearance upon smiling. The following are specific diagnostic observations:

1. BC‐CP: greater than ideal due to severe bone loss which was accompanied by apical migration of the gingival margin.
2. Interproximal width: excessive presence of a diastema between the central and lateral incisor.
3. Gingival thickness: generally adequate, but increasing gingival thickness could contribute to achieving a greater papilla height.
4. Tooth form: ideal, square shape.
5. Axial root inclination: abnormal mainly due to pathologic tooth migration.

Other than the tooth form, all other factors were unfavorable, and the patient presented “active” periodontitis, which took priority. After initial periodontal and endodontic therapy (central incisor), periodontal surgery was performed to improve BC‐CP by attempting to reconstruct both infra‐ and supra‐alveolar defects (SAPRE technique). Additionally, soft tissue augmentation was done to increase the gingival thickness (Figure 6A–F, Table 1).

**TABLE 1:** Scenario 4: unhealthy periodontal tissues. Clinical parameters, probing pocket depth (PD) and clinical attachment level (CAL), were measured just before the surgery (baseline) and at 4 years post‐surgery.

	Baseline and postoperative clinical parameters					
	Tooth	12			11	
	Site	Distal	Central	Mesial	Distal	Central
PD labial (mm)	Baseline	7	2	7	7	5
	4 years	3	3	4	4	3
	Change	4	−1	3	3	2
CAL labial (mm)	Baseline	12	7	12	11	10
	4 years	4	3	5	5	3
	Gain	8	4	7	5	7

As a result, there was an esthetic improvement in the papilla and a reduction in gingival recession compared to the preoperative state. Although orthodontic treatment was recommended to improve interproximal width and axial root inclination of the lateral incisor, the patient declined it. The result remained stable over a 4‐year postoperative period (Figure 6).

## 5 Discussion

For an optimal esthetic outcome, achieving harmony between the white (tooth structure) and pink (soft tissue) is crucial. This requires not only an adequate volume and positioning of both periodontal hard and soft tissues, but also an ideal tooth form and proper alignment of the dental roots. These interdependent factors collectively contribute to achieving a pleasing smile.

In the presence of OIE, treatment is often driven by the patient's esthetic concerns, although functional issues may also be present. Numerous reports have documented the esthetic and functional implications of black triangles; yet individualized care remains essential [45, 46, 47]. It is the healthcare team members' responsibility to present all viable options and tailor the treatment plan to the patient's specific needs and expectations.

In this article, a set of key etiologic and risk factors that should be accounted for in the diagnostic and treatment planning phase, as well as a systematic decision‐making framework for managing OIE are outlined. As emphasized, comprehensive diagnosis and sound treatment sequencing are paramount. A critical consideration is to distinguish between esthetic demands and pathological entities. Naturally, when the presence of an inflammatory disease such as periodontitis is identified, it must take precedence in the treatment sequence, as illustrated in Scenario 4.

When treating the sequelae of periodontitis or structural deformities caused by other pathological processes, regenerative therapies that target both hard and soft tissue augmentation, such as the SAPRE technique, are increasingly recognized for their potential to positively influence papillary morphology. Soft tissue augmentation involving the use of CTG, for instance, has demonstrated synergistic effects in improving both gingival thickness and underlying bone regeneration [48, 49].

However, in cases with severe periodontal breakdown and wide interproximal spaces, such as Scenario 4, even with stable regenerative outcomes, esthetic limitations like persistent “black triangles” may remain. In such situations, clinicians should carefully weigh the esthetic prognosis and overall tooth restorability. Extraction of severely compromised teeth may be a valid option when esthetic demands are high. The resulting edentulous space can be managed with dental implants or fixed dental prostheses that incorporate prosthetic gingival reconstruction to mask hard and soft tissue deficiencies. These options may offer more predictable and satisfactory esthetic outcomes in select cases where regenerative or orthodontic interventions alone cannot fully resolve the papillary deficiencies.

As illustrated in Scenario 2, orthodontic therapy remains a reliable modality not only for repositioning teeth, but also to enhance soft tissue esthetics by modifying root angulation and contact point position. While orthodontic movement may occasionally result in papillary recession or soft tissue remodeling [4, 5], its strategic application can significantly enhance gingival contour and papilla height [36].

Restorative and prosthetic interventions are equally important therapeutic resources but must be planned and executed with meticulous attention to detail. It is well established that suboptimal restorations can compromise periodontal health and esthetics [50], while well‐executed restorative treatments can enhance the overall appearance and function of the dentition [47]. Thoughtful integration of “pink esthetics” into prosthetic planning is essential in modern restorative dentistry, especially in anterior regions where esthetic demands are highest.

## 6 Final Remarks

Effective and predictable management of OIE often requires a nuanced and well‐articulated interdisciplinary approach, including a thorough understanding of the key interrelated factors and treatment modalities addressed in this article. Accurate diagnosis and treatment sequence planning accounting for these variables are fundamental to achieving stable, esthetic, and functional outcomes. The integration of periodontal, orthodontic, and prosthetic strategies tailored to the individual patient's needs is the cornerstone of successful treatment.

The therapeutic principles hereby discussed mainly apply to the interproximal areas in the natural dentition, but may also be interpolated to restorative interfaces between teeth and pontics, teeth and dental implants, and between adjacent dental implants.

Future studies aimed at evaluating the reliability and applicability of the concepts and clinical recommendations hereby presented are warranted.

## Conflicts of Interest

The authors declare no conflicts of interest.

## Data Availability

The data that support the findings of this study are available from the corresponding author upon reasonable request.
